# Turpentine in Dysentery

**Published:** 1852-06

**Authors:** John Long

**Affiliations:** Pleasantville, Ky.


From the St. Louis Med. and Surg. Journal.

*Turpentine in Dysentery.* By JOHN LONG, M. D., of Pleasantville, Ky.

FOR more than twelve months past, I have been in the habit of using turpentine in the treatment of dysentery, as it has occurred in this section of country, and find it to be a most valuable remedy in this often formidable disease. I have employed it in cases where the irritation or inflammation seemed to be confined to the lower portion of the bowels, as well as such as were complicated with typhoid fever. Dose, ten drops of the turpentine combined with twenty drops of laudanum, for an adult, every eight hours—with mucilagenous drinks and farinaceous diet. In obstinate cases it is necessary, in conjunction with the above, to resort to the ordinary enemata of laudanum and starch. Other remedies, as mercury, quinine or astringents, may also be used as circumstances require.

I was first induced to resort to turpentine in the treatment of dysentery, at the suggestion of Dr. Wood, of Philadelphia, who recommends it in typhoid fever.

During the past summer I treated thirty cases according to the above method, twenty-nine of which recovered and one died; the latter resided fifteen miles off, and I did not see him but once.

